# Independent risk factors associated with the decision to withhold therapeutic intervention in patients admitted to the emergency room

**DOI:** 10.1186/cc13226

**Published:** 2014-03-17

**Authors:** L Serpa-Pinto, T Cardoso

**Affiliations:** 1 Oporto Hospital Center, Oporto, Portugal

## Introduction

The decision to withhold therapeutic intervention in a patient is a complex decision, wrapped in profound ethical debates. The role of the physician is to cure, treat and alleviate suffering. When the first two goals are not possible the medical role should be dedicated to end-of-life treatments adjusted to the patient's benefit [1,2].

## Methods

A retrospective cohort study including all adult patients with sepsis admitted to the emergency room (ER) at a tertiary care, university hospital between 1 July 2011 and 30 June 2012.

## Results

During the study period 162 patients with sepsis were admitted to the ER, of which 40 (25%) had withheld therapeutic decisions. Comparing this group with the group without therapeutic limitations, patients in the first group were older (81 ± 13 vs. 68 ± 14, *P *< 0.001), with more comorbidities (90% vs. 66%, *P *= 0.004) and a higher proportion needing help in daily activities (Karnofsky performance status (KPS) < 70% = 55% vs. 8%, *P *< 0.001). The hospital mortality in patients with a decision to limit the therapeutic intervention was significantly higher (83% vs. 43%, *P *< 0.001). Variables independently associated with the withholding therapy decision were age (adjusted OR per year = 1.078, *P *< 0.001), presence of comorbidities (adjusted OR = 4.632, *P *= 0.030), chronic wounds (adjusted OR = 5.965, *P *= 0.005) and patient's needed of help in daily activities (KPS < 70%, adjusted OR = 5.391, *P *= 0.012). In the first group a lower proportion received antibiotics (70% vs. 99%, *P *< 0.001) and when those were considered inadequate for the agent responsible for the sepsis episode it was less frequently changed (15% vs. 50%, *P *= 0.028). However, no differences were found regarding the elapsed time from admission to the ER until the first medical contact or the time since the recognition of sepsis and antibiotic administration, although the group with withholding decisions had less specimens collected for microbiology: blood cultures (68% vs. 91%, *P *< 0.001) or other specimens (58% vs. 96%, *P *< 0.001).

## Conclusion

In this study the decision to withhold therapy was independently associated with increasing age, the presence of comorbidities and loss of functional autonomy. For the same level of intervention such as antibiotic administration, the decision to withhold therapy did not influence the efficacy of therapeutic attitudes.
